# Anti-Inflammatory Effect of Chestnut Honey and Cabbage Mixtures Alleviates Gastric Mucosal Damage

**DOI:** 10.3390/nu16030389

**Published:** 2024-01-29

**Authors:** Hyo-Jung Kim, Bo-Ram Jin, Chang-Dae Lee, Doyun Kim, Ah Young Lee, Sanghyun Lee, Hyo-Jin An

**Affiliations:** 1 Department of Oriental Pharmaceutical Science, College of Pharmacy, Kyung Hee University, Seoul 02447, Republic of Korea; hyojung_95@naver.com (H.-J.K.); wlsqh92@khu.ac.kr (B.-R.J.); 2 Department of Plant Science and Technology, Chung-Ang University, Anseong 17546, Republic of Korea; great7321@naver.com; 3 KEDEM Inc., Chuncheon-si 24341, Republic of Korea; doyun325@naver.com; 4 Department of Food Science, Gyeongsang National University, Jinju 52725, Republic of Korea; aylee@gnu.ac.kr; 5 Department of Integrated Drug Development and Natural Products, Graduate School, Kyung Hee University, Seoul 02447, Republic of Korea

**Keywords:** gastritis, indomethacin, inflammation, oxidative stress

## Abstract

Gastritis, one of the most common gastrointestinal disorders, damages the stomach lining as it causes a disproportion between the protective and ruinous factors of the gastric system. Cabbage (CB) is widely used to treat gastric lesions but requires the addition of natural sweeteners to counteract its distinct bitter taste. Therefore, this study sought to determine whether the combination of chestnut honey (CH)—which is known for its dark brown color and high kynurenic acid (KA) content—or KA-increased CH (KACH) with CB (CH + CB or KACH + CB) exerts synergistic effects for improving both taste and efficacy. Before confirming the gastroprotective effects in indomethacin (INDO)-induced rats, the anti-inflammatory activities of CH + CB and KACH + CB were assessed in lipopolysaccharide (LPS)-stimulated RAW 264.7 macrophages. As a result, treatment with either CH + CB or KACH + CB downregulated pro-inflammatory cytokine levels in LPS-stimulated RAW 264.7 macrophages by regulating the translocation of nuclear factor kappa B. Furthermore, both CH + CB and KACH + CB not only enhanced the levels of antioxidant enzymes but also triggered the activation of nuclear factor erythroid-related factor 2. Based on these effects, CH + CB or KACH + CB effectively protected the gastric mucosa in INDO-induced rats. Therefore, this study suggests that CH + CB and KACH + CB exert stronger gastroprotective effects when used together.

## 1. Introduction

Gastritis—characterized by ulcers, haemorrhaging, and erosion of the gastric mucosa —is a major digestive disorder that is increasing in prevalence worldwide [1]. Gastritis is mainly caused by multiple factors—such as *Helicobacter pylori* infection, poor dietary habits, stress, excessive drinking, and prolonged use of nonsteroidal anti-inflammatory drugs (NSAIDs) [2], which are the most frequently prescribed drugs for inflammatory diseases given their antipyretic and analgesic effects; however, they can also damage the gastrointestinal tract [3]. Although drugs such as omeprazole (a proton pump inhibitor) and misoprostol (a synthetic analogue of prostaglandin E; PGE) are used to prevent and treat gastritis caused by NSAIDs, they have certain limitations and various side effects [4]. Therefore, to discover alternative agents for the treatment of gastritis, it is necessary to identify natural products with fewer adverse effects and for mechanistic studies.

Inflammation and oxidative stress, which are intimately connected cellular processes, contribute to the development of NSAID-induced gastric mucosal damage by interacting with each other [5]. The excessive generation of reactive oxygen species (ROS) not only triggers lipid peroxidation to improve gut permeability but also stimulates macrophages, promoting the activation of inflammatory transcription factors, such as nuclear factor kappa B (NF-κB) [6]. This triggers a vicious cycle by releasing pro-inflammatory cytokines, such as interleukin (IL)-6, IL-1β, and tumor necrosis factor-α (TNF-α), that contribute to ROS production [7]. On the other hand, nuclear factor erythroid-related factor 2 (Nrf2) serves as a key transcriptional regulator of antioxidant enzymes, including heme oxygenase 1 (HO-1), glutathione peroxidase 1 (GPx1), superoxide dismutase 1 (SOD1), and glutathione S-transferase (GST) superfamily, preserving cells from oxidative stress mediated by inflammatory signals [8,9]. From this point of view, Nrf2 is considered an essential therapeutic target that can alleviate gastritis by regulating inflammation and oxidative stress.

Cabbage (CB; *Brassica oleraceae* var. *capitata* L.), which belongs to the Brassicaceae family, has been widely utilized to treat gastrointestinal disorders owing to its excellent anti-inflammatory and antioxidant properties [10,11,12]. Despite these health-enhancing effects, glucosinolates—which contribute to the bitterness of CB—negatively affect consumer preferences [13]. Therefore, sweeteners are thought to facilitate consumption by masking CB bitterness. Honey, which is widely used as a natural sweetener, is a supersaturated sugar solution produced by honeybees using nectar and honeydew secreted from the exudates of plants and trees [14]. Chestnut honey (CH), produced from the flowers of the chestnut tree (*Castanea crenata* Siebold & Zucc.), is characterized by a dark color and is not easily crystallized [15]. CH, which is rich in polyphenols, has been useful in the treatment of inflammation-related chronic disorders because of its strong antioxidant action [16]. Additionally, previous studies have reported that CH contains a substantial amount of kynurenic acid (KA), a metabolite of tryptophan with potential anti-inflammatory properties [17,18,19,20]. Therefore, increasing the KA content in CH (KACH) may be a crucial factor for treating inflammation. Despite the efficacy of CH and KACH, which are both suitable treatments for gastritis, no studies have focused on their combination with CB. In this regard, the study sought to confirm whether the combination of CH or KACH with CB (CH + CB or KACH + CB) not only enhances anti-inflammatory effects but also alleviates gastric mucosal damage. To achieve this, RAW 264.7 macrophages were stimulated with lipopolysaccharide (LPS), an endotoxin of Gram-negative bacteria known to activate macrophages, and rats were administered with indomethacin (INDO), an NSAID that induces gastritis.

## 2. Materials and Methods

### 2.1. Chemicals and Antibodies

Inducible nitric oxide synthase (iNOS; M00368) was used as a primary antibody from Boster Biological Technology (Pleasanton, CA, USA). Inhibitors of κB-α (IκB-α; sc-203), IκB kinase-α/β (IKK-α/β; sc-7607), HO-1 (sc-136960), and β-actin (sc-47778) were used as primary antibodies (Santa Cruz Biotechnology, Inc., Dallas, Texas, USA). NF-κB p65 (4764), phospho-IκB-α (9246), phospho-IKK-α/β (Ser176/180; 2697), kelch-like ECH-associated protein 1 (keap1; sc-514914), and poly ADP-ribose polymerase (PARP; 9542) were used as primary antibodies (Cell Signaling Technology, Danvers, MA, USA). Nrf2 (ab92946) was used as a primary antibody (Abcam, Cambridge, UK). Horseradish peroxidase-conjugated (HRP) secondary antibodies were obtained from Jackson ImmunoResearch (West Grove, PA, USA). Commercial enzyme-linked immunosorbent assay (ELISA) kits for PGE2, myeloperoxidase (MPO), and SOD were purchased from R&D Systems (Minneapolis, MN, USA), Biovision (Milpitas, CA, USA), and Abcam (Cambridge, MA, USA). Unless otherwise specified, all remaining reagents were purchased from Sigma-Aldrich (St. Louis, MO, USA).

### 2.2. Preparation of Samples

CH originating from “Damyang” was used to establish the optimal extraction method for the validation of CH; the effects of different solvent compositions, solvent volumes, and extraction times were investigated under reflux extraction conditions. A 5 g honey sample was extracted using 100% water and 10% or 30% EtOH in volumes of 50 mL and 100 mL, which were 10 and 20 times the sample volume, respectively. Samples were extracted at 3 or 6 h. In addition to honey extracts, unextracted and freeze-dried CH samples were analyzed, and their respective KA contents were compared. After the extracted sample was filtered, it was placed in an oven (55 °C) for 1 d and freeze-dried to obtain freeze-dried extracts. CB (2.1 kg) was boiled in water (900 mL) in a 100 °C water bath for 3 h and then pressed. Finally, approximately 1.8 L of the product was obtained and dried using a freeze dryer (FDU-1200, Tokyo, Japan, EYELA). Stillen^®^ (STI), used for the treatment of gastritis and peptic ulcers in Asian countries, including the Republic of Korea [21], was purchased from Dong-A ST Co., Ltd. (Seoul, Republic of Korea) and utilized as a positive control.

### 2.3. High-Performance Liquid Chromatography-Variable Wavelength Detector Analysis

Quantitative analyses were conducted using a reversed-phase HPLC (RP-HPLC) coupled with a VW detector (Agilent 1260 Infinity II Quat Pump, Santa Clara, CA, USA). KA and sinapic acid (SA), the standards for CH and CB, respectively, were acquired from the Natural Product Institute of Science and Technology (Anseong, Gyeonggi-do, Republic of Korea). CH and CB single or in combination (MCHCB) 1–4 (MCHCB 1: CH, MCHCB 2: CB, MCHCB 3: CH + CB 1:9, and MCHCB 4: KACH + CB 1:9; 250 mg), KA (1 mg), and SA (1 mg) were dissolved in 70% methanol (1 mL) and sonicated for 20 min. After that, all samples were filtered using a PTFE membrane syringe filter (pore size 0.45 μm) to prepare the stock solutions of samples. Quantitative analysis was performed using an RP-HPLC with a YMC Pack Pro C_18_ column (4.6 mm × 25 cm, 5 μm). The column oven was set to 30 °C and an auto-sampler was set to room temperature. The wavelength for VWD was set at 240 nm and sample injection volume was 10 μL. The analysis employed a gradient elution system, utilizing a mobile phase composed of 0.1% trifluoroacetic acid in distilled water (solvent A) and acetonitrile (solvent B), with a total runtime of 55 min. The gradient elution conditions of the mobile phase began with 95% of solvent A, which was gradually reduced to 82% over 10 min and maintained for 10 min. Subsequently, solvent A was reduced to 60% after 10 min. The amount of solvent B was then increased to 100% for 5 min and kept for 5 min. Finally, the concentration of solvent A was increased to 95% after 5 min and was maintained for 10 min.

### 2.4. Calibration Curve

KA and SA were diluted to different concentrations (0.0625–1.0000 mg/mL and 0.0009–0.0156 mg/mL, respectively) to prepare calibration curves for quantitative analysis. A calibration curve was obtained by plotting the area of each detected peak according to the concentration, with the correlation coefficient (*r*^2^) used to prove the accuracy and linearity of the calibration curve. The concentrations of KA and SA, the standard substances contained in MCHCB 1–4, were calculated using the obtained calibration curves. The calibration functions for KA and SA were determined by setting the *X*-axis to the sample concentration (mg/mL) and the *Y*-axis to the detected peak area.

### 2.5. Cell Culture and Cell Viability Assay

Murine RAW 264.7 macrophages were procured from the Korea Cell Line Bank (Seoul, Republic of Korea). Macrophages were cultivated in Dulbecco’s modified eagle’s medium, which was supplemented with 10% fetal bovine serum and 1% penicillin-streptomycin in a 37 °C humidified incubator maintained at 5% CO_2_ (Life Technologies, Grand Island, NY, USA). To determine the cell viability, macrophages seeded at a concentration of 1 × 10^5^ cells per well were treated with 0–100 μg/mL samples for 24 h. Then, 3-(4,5-Dimethyl-2-thiazolyl)-2,5-diphenyl-2H-tetrazolium bromide (MTT; 5 mg/mL) reagent was added and further incubated in a 37 °C humidified incubator for 4 h. After inhaling the supernatant, the remaining insoluble formazan was dissolved using dimethyl sulfoxide. Absorbance was determined at 540 nm using the microplate spectrophotometer (Winooski, VT, USA).

### 2.6. Nitric Oxide (NO) Assay

To determine the NO production, RAW 264.7 macrophages seeded at a concentration of 1 × 10^5^ cells per well were pretreated with samples (0–100 μg/mL) and L-N6-(1-Iminoethyl)lysine (NIL; 20 μM) for 1 h before stimulation with LPS (1 μg/mL). After 48 h, 100 μL of the collected culture medium for each sample was reacted with 100 μL of Griess reagent mixed with 1% sulfanilamide in 5% phosphoric acid and 1% α-naphthylamide in H_2_O at a 1:1 ratio. NO production was then determined at 540 nm using the microplate spectrophotometer (Winooski, VT, USA).

### 2.7. Western Blot Analysis

Pure protein was extracted from macrophages using a protein extraction solution (PRO-PREP™; iNtRON Biotechnology, Seongnam, Gyeonggi-do, Republic of Korea) following the producer’s guidelines. Protein concentration was determined using Bradford assay dye (Bio-Rad, Hercules, CA, USA). Each protein was applied to an 8–12% sodium dodecyl sulfate-polyacrylamide gel and transferred to a polyvinylidene fluoride membrane. Membranes coated with skim milk (1–5%) were exposed to diluted primary antibodies overnight at 4 °C. Immunoreactive proteins were identified utilizing an enhanced luminol-based detection reagent (GE Healthcare Life Sciences Inc., Chicago, IL, USA) in a membrane reacted with HRP secondary antibodies for 2 h.

### 2.8. Separation of Cytoplasmic and Nuclear Proteins

RAW 264.7 macrophages were resuspended by adding a hypotonic buffer containing phenylmethylsulfonyl fluoride (PMSF; 0.2 mM), aphlotin (10 μg/mL), dithiotratol (DTT; 0.5 mM), KCl (10 mM), HEPES (10 mM, pH 7.9), and MgCl2 (1.5 mM). To obtain the cytoplasmic fraction, 0.1% Nonidet P-40 was added, and the supernatant was stored by microcentrifugation at 15,920× *g*. Subsequently, the remaining pellets were resuspended in high-salt buffer containing sodium orthovanadate (1 mM), DTT (0.5 mM), glycerol (25%), NaF (1 mM), EDTA (0.2 mM), KCl (400 mM), HEPES (20 mM, pH 7.9), and MgCl2 (1.5 mM). To obtain the nuclear fraction, the supernatant was stored by microcentrifugation at 15,920× *g*.

### 2.9. Quantitative Real-Time-Polymerase Chain Reaction (qRT-PCR)

According to the producer’s guidelines, total RNA was extracted from RAW 264.7 macrophages using easy-BLUE™ reagent (iNtRON Biotechnology, Inc., Seongnam, Gyeonggi-do, Republic of Korea). The total RNA was determined using a microplate spectrophotometer (Winooski, VT, USA) and then reverse transcribed into cDNA. Oligonucleotide primers of IL-6, TNF-α, HO-1, SOD1, GPx1, GSTM1, GSTP1, and β-actin were manufactured by Bioneer Corporation (Daejeon, Republic of Korea), and their sequences are detailed in Table 1. Relative gene expression was detected with PowerUp™ SYBR^®^ green master mix using Real-Time PCR System 7500 (Applied Biosystems, Foster, CA, USA). Equation 2^−ΔΔCt^ was utilized to calculate the relative gene expression, and β-actin was used for normalization.

### 2.10. Experimental Animals

Seven-week-old male Sprague Dawley rats were provided by Koatech (Pyeongtaek, Gyeonggi-do, Republic of Korea). The rats were acclimatized for one week under constant conditions (temperature: 23 ± 3 °C; humidity: 55 ± 15%; light cycle: 12 h). To conduct animal experiments, approval was obtained from the Institutional Animal Care and Use Committee at KNOTUS Co, Ltd. (Guri, Gyeonggi-do, Republic of Korea; KNOTUS IACUC 22-KE-0129 & 21-KE-0219). For the first experiment, rats were segmented into the following eight groups (*n* = 5 per group): control (CON), INDO, STI, CH, CB, and CH + CB (1:9, 1:1, and 9:1). STI (100 mg/kg), CB (4.5 g/kg), CH (0.5 g/kg), CH + CB 1:9 (0.5 + 4.5 g/kg), CH + CB 1:1 (2.5 + 2.5 g/kg), and CH + CB 9:1 (4.5 + 0.5 g/kg) were orally administered to rats that had been fasted for 48 h, with a total dose of 5 mL/kg. After 30 min, all rats, except those in the CON group, were orally administered INDO (80 mg/kg) dissolved in sodium bicarbonate to induce gastritis. After 5 h, the rats were sacrificed under ether anaesthesia, and their stomachs were surgically removed. For the second experiment, rats were segmented into the following nine groups (*n* = 8–10 per group): CON, INDO (80 mg/kg), STI (100 mg/kg), CH (1.5 g/kg), KACH (1.5 g/kg), CB (13.5 g/kg), CH + CB (15 g/kg), KACH + CB (15 g/kg), and KA (1.5 mg/kg). The total dosage was 15 mL/kg, and the experimental method was the same as that used in the first experiment.

### 2.11. Histological Analysis

The gastric mucosa of each rat was rapidly removed and cut along the greater curvature. The sides of the gastric mucosa were photographed using a digital camera. The damaged area of the gastric mucosa was calculated as a percentage (%) of the damaged area compared to the total gastric area using ImageJ software (National Institutes of Health, Bethesda, Maryland, USA). The tissue was immediately fixed in 10% formalin and then embedded in paraffin. Paraffin sections were sliced with a thickness of 4 μm by using a microtome and stained with hematoxylin and eosin (H&E; BBC biochemical, Berno, WA, USA); the haemorrhage and erosion were observed microscopically. Histological scores were used to evaluate tissue damage, as previously described [22], as follows: 0 = no damage; 1 = minimal damage; 2 = mild damage; 3 = moderate damage; 4 = severe damage.

### 2.12. ELISA Assay

Gastric mucosa tissues were homogenized in cold-PBS using a bead homogenizer (Omni-Inc., Kennesaw, GA, USA) at 5000× *g* for 5 min at 4 °C. The supernatants were subsequently utilized to measure the levels of PGE_2_, MPO, and SOD by using commercial ELISA kits according to the producer’s guidelines.

### 2.13. Statistics

The experimental values are depicted as the mean ± standard deviation (SD) from triplicate experiments. Significant differences were evaluated via a one-way analysis of variance followed by Dunnett’s post hoc test using GraphPad Prism software (version 5.01, San Diego, CA, USA). Values with a *p* less than that of 0.05 were considered to have statistically significant differences (* *p* < 0.05, ** *p* < 0.01, *** *p* < 0.001).

## 3. Results

### 3.1. Quantitative Analysis Results of MCHCB for KA and SA

To assess the content of bioactive components in MCHCB according to the mixing ratio, HPLC-VWD analysis was conducted using standardized compounds of KA and SA. The calibration curves exhibited exceptional linearity with *r*^2^ values of 0.9990 and 0.9992 for KA and SA, respectively (Figure S1A, Table S1). The KA and SA contents of MCHCB 1–4 were determined relative to the mixing ratio. For SA compounds, trace amounts were detected in MCHCB 2, 3, and 4. For the KA compound, the highest level was detected in MCHCB 1. In the case of mixtures with CH and KACH to CB ratio of 1:9, MCHCB 4 exhibited a higher KA content than MCHCB 3 (Figure S1B–E, Table S2). These results affirm the successful preparation of KACH with an elevated KA content in CH.

### 3.2. CH and CB Mixtures Suppress the Expression of NO and iNOS in LPS-Stimulated RAW 264.7 Macrophages

**Figure 1:**
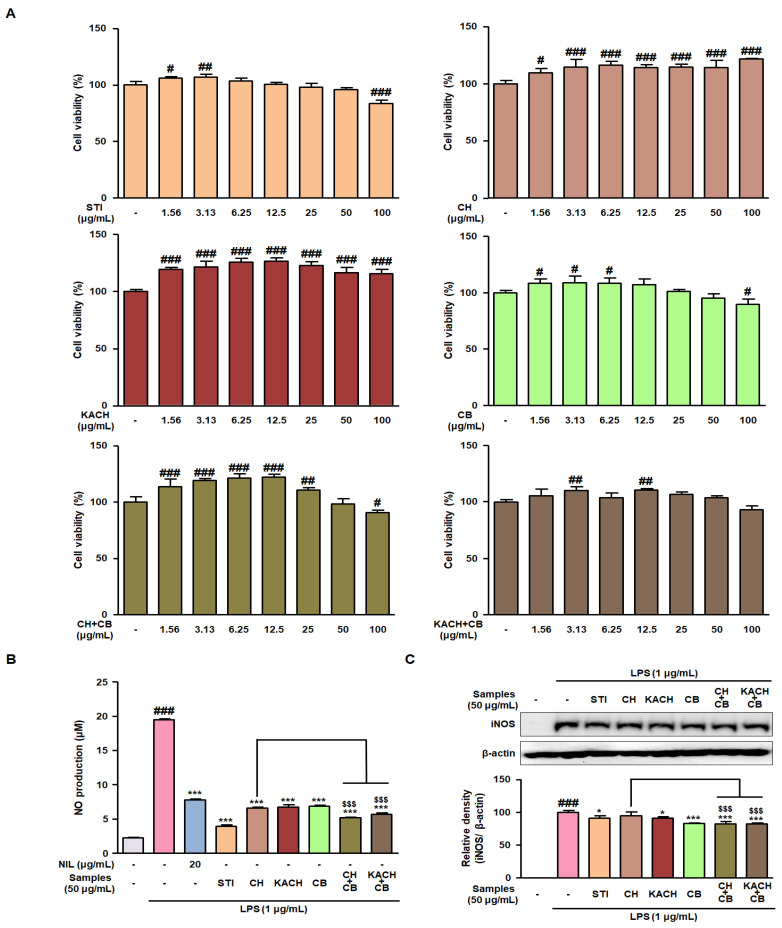
Inhibitory effect of CH and CB mixtures on NO production and iNOS protein expression in LPS-stimulated RAW 264.7 macrophages. (**A**) The survival rate of RAW 264.7 macrophages treated with various concentrations (0–100 μg/mL) of STI, CH, KACH, CB, CH + CB, and KACH + CB was determined by MTT assay. ^#^ *p* < 0.05, ^##^ *p* < 0.01, ^###^ *p* < 0.001 vs. the non-stimulated macrophages. (**B**) Culture supernatants of LPS-stimulated RAW 264.7 macrophages treated with STI, CH, KACH, CB, CH + CB, and KACH + CB were reacted with a Griess reagent to measure NO levels. (**C**) iNOS protein expression was detected by Western blotting using specific antibodies. Relative density was expressed by normalizing to an internal control, β-actin. Results are presented as mean ± SD of three independent experiments. ^###^ *p* < 0.001 vs. the non-stimulated macrophages; * *p* < 0.05, *** *p* < 0.001 vs. the LPS-stimulated macrophages. ^$$$^ *p* < 0.001 vs. the CH-treated macrophages. LPS, lipopolysaccharide; NIL, L-N6-(1-Iminoethyl)lysine; STI, Stillen^®^; CH, chestnut honey; KACH, kynurenic acid increased CH; CB, cabbage; CH + CB, a mixture of CH and CB; KACH + CB, a mixture of KACH and CB.

Before confirming whether mixing the two types of CH with CB has a greater anti-inflammatory effect than that of a single treatment, an MTT assay was conducted to unify the treatment concentration of the samples. In RAW 264.7 macrophages, a significant decrease in cell viability was observed only at a concentration of 100 μg/mL in treatments with STI, CB, and CH + CB (Figure 1A). Based on this, the treatment concentration for subsequent bioassay was set to 50 μg/mL, a concentration that does not exhibit cytotoxicity. The culture supernatant of RAW 264.7 macrophages stimulated with LPS was reacted with the Griess reagent to investigate whether NO production was reduced by sample treatment. The elevated NO levels stimulated by LPS were significantly reversed by sample treatment and exhibited a better inhibitory effect than NIL, a known selective iNOS inhibitor (Figure 1B). In particular, treatment with CH + CB and KACH + CB reduced NO levels to the same extent as the positive control (STI). In addition, iNOS protein expression, which was markedly upregulated by LPS stimulation, was slightly downregulated by CH + CB and KACH + CB treatment (Figure 1C).

### 3.3. CH and CB Mixtures Suppress the mRNA Expression of Pro-Inflammatory Cytokines by Modulating NF-κB Activity in LPS-Stimulated RAW 264.7 Macrophages

**Figure 2:**
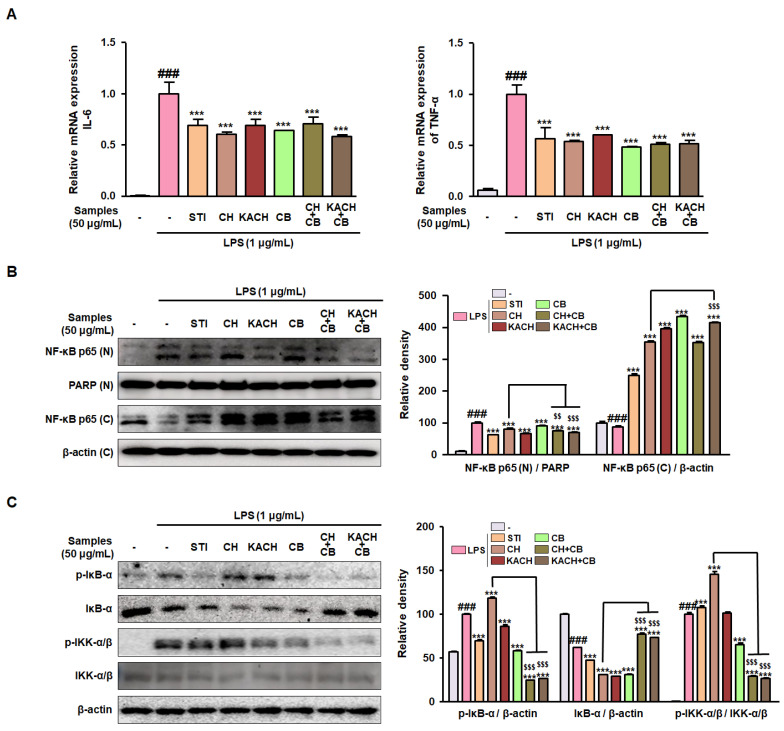
Inhibitory effect of CH and CB mixtures on mRNA expression of pro-inflammatory cytokines through regulating NF-κB activation in LPS-stimulated RAW 264.7 macrophages. (**A**) IL-6 and TNF-α mRNA expressions were determined using qRT-PCR. Results were expressed by normalizing to internal control, β-actin. (**B**) NF-κB p65 protein expression in the nuclear and cytoplasmic fractions was detected by Western blotting using specific antibodies. (**C**) p-IκB-α, IκB-α, p-IKK-α/β, and IKK-α/β protein expressions were detected by Western blotting using specific antibodies. Relative density was expressed by normalizing it with PARP or β-actin, an internal control of nuclear or cytoplasmic fractions. Results are presented as mean ± SD of three independent experiments. ^###^ *p* < 0.001 vs. the non-stimulated macrophages; *** *p* < 0.001 vs. the LPS-stimulated macrophages. ^$$^ *p* < 0.01, ^$$$^ *p* < 0.001 vs. the CH-treated macrophages. LPS, lipopolysaccharide; STI, Stillen^®^; CH, chestnut honey; KACH, kynurenic acid increased CH; CB, cabbage; CH + CB, a mixture of CH and CB; KACH + CB, a mixture of KACH and CB.

To confirm whether the mixing of CH and CB modulates the pro-inflammatory cytokine expression, IL-6 and TNF-α mRNA expressions were examined. The mRNA expression of IL-6 and TNF-α elevated by LPS stimulation was decreased by STI, CH, KACH, CB, CH + CB, and KACH + CB treatment in RAW 264.7 macrophages (Figure 2A). To examine whether the anti-inflammatory effect of CH and CB mixtures is exerted by blocking the transcriptional activity of the NF-κB signalling pathway, the relevant markers were detected. Nuclear protein expression of NF-κB p65 was overexpressed by LPS stimulation, which was reduced by treatment with STI, CH, KACH, CB, CH + CB, and KACH + CB. In contrast, the protein expression of NF-κB p65 in the cytoplasm, which was downregulated by LPS stimulation, was restored by STI, CH, KACH, CB, CH + CB, and KACH + CB (Figure 2B). As a result of confirming the phosphorylation of IκB-α and IKK-α/β, which are upstream of the NF-κB signalling pathway, the protein expressions of p-IκB-α and p-IKK-α/β increased by LPS stimulation were significantly suppressed in CH + CB and KACH + CB treatments than in single treatments (Figure 2C). These results demonstrate that the treatment with CH + CB and KACH + CB effectively blocks the nuclear translocation of the NF-κB signalling pathway, leading to the suppression of inflammatory responses, compared to the single treatments with CH or CB.

### 3.4. CH and CB Mixtures Enhance Antioxidant Enzymes by Modulating Nrf2 Activity in LPS-Stimulated Raw 264.7 Macrophages

**Figure 3:**
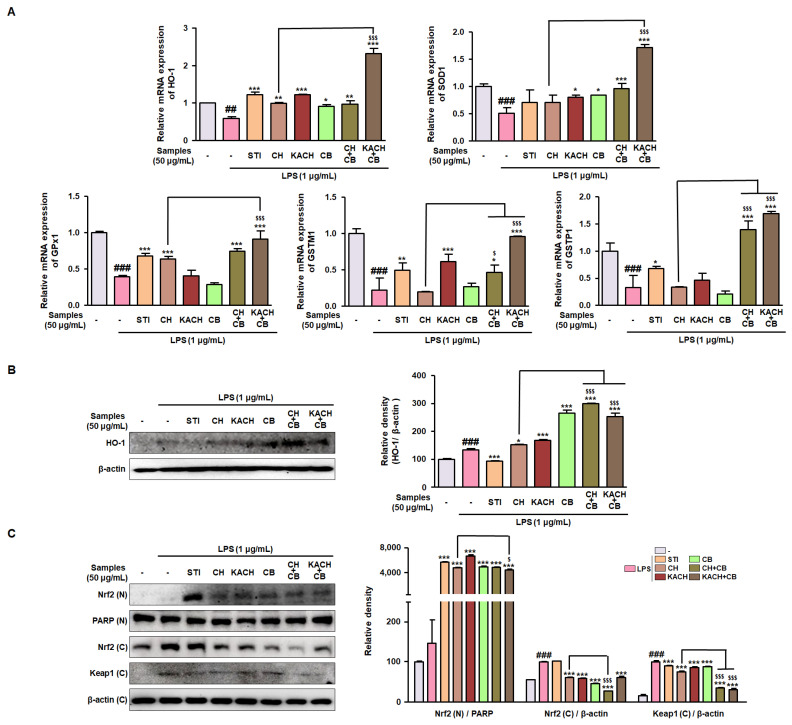
Enhancing effect of CH and CB mixtures on mRNA expression of antioxidant enzymes through regulating Nrf2 activation in LPS-stimulated RAW 264.7 macrophages. (**A**) HO-1, SOD1, GPx1, GSTM1, and GSTP1 mRNA expressions were determined using qRT-PCR. Results are expressed by normalizing to an internal control, β-actin. (**B**) HO-1 protein expression was detected by Western blotting using specific antibodies. (**C**) Nrf2 and Keap1 protein expressions in the nuclear and cytoplasmic fractions were detected by Western blotting using specific antibodies. Relative density was expressed by normalizing it with PARP or β-actin, an internal control of nuclear or cytoplasmic fractions. Results are presented as mean ± SD of three independent experiments. ^##^ *p* < 0.01, ^###^ *p* < 0.001 vs. the non-stimulated macrophages; * *p* < 0.05, ** *p* < 0.01, *** *p* < 0.001 vs. the LPS-stimulated macrophages. ^$^ *p* < 0.05, ^$$$^ *p* < 0.001 vs. the CH-treated macrophages. LPS, lipopolysaccharide; STI, Stillen^®^; CH, chestnut honey; KACH, kynurenic acid increased CH; CB, cabbage; CH + CB, a mixture of CH and CB; KACH + CB, a mixture of KACH and CB.

To demonstrate whether mixing of CH and CB inhibits oxidative damage stimulated by LPS, the mRNA expression of antioxidant enzyme-related genes was measured by qRT-PCR. In LPS-stimulated RAW 264.7 macrophages, downregulated mRNA expression of HO-1, SOD1, GPx1, GSTM1, and GSTP1 was significantly improved by treatment with the combination of CH and CB, especially KACH + CB (Figure 3A). This effectiveness surpasses that of STI, known for exerting antioxidant activity by promoting the nuclear translocation of Nrf2 [23]. Accordingly, the Nrf2/HO-1 signalling pathway was investigated to elucidate the molecular mechanism underlying the protective effect of CH + CB and KACH + CB against oxidative damage. In LPS-stimulated RAW 264.7 macrophages, CH, KACH, CB, CH + CB, and KACH + CB treatments significantly enhanced the diminished HO-1 protein expression (Figure 3B). To confirm the nuclear translocation of Nrf2, its protein expression was detected in the nuclear and cytoplasmic fractions obtained from LPS-stimulated RAW 264.7 macrophages. The protein expression of Nrf2 in the nucleus, which was suppressed by LPS stimulation, was restored by STI, CH, KACH, CB, CH + CB, and KACH + CB. The protein expression of Nrf2 and Keap1 in the cytoplasm was elevated by LPS stimulation, whereas treatment with STI, CH, KACH, CB, CH + CB, and KACH + CB attenuated this effect. These results indicate that the combination of CH and CB exerts antioxidant effects by regulating the Nrf2 signalling pathway.

### 3.5. Various Ratios of CH and CB Mixtures Protect against Gastric Damage in INDO-Induced Rats

**Figure 4:**
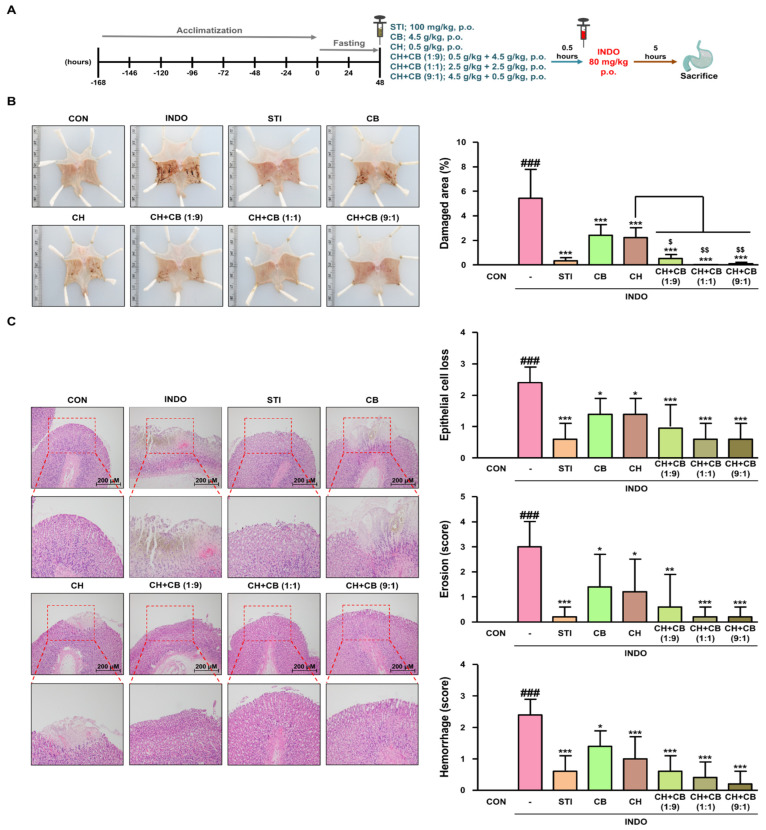
The protective effect of CH and CB mixtures at various ratios on gastric damage in INDO-induced rats. (**A**) Schematic diagram of the first animal experiment. (**B**) Visual inspection of gastric lesions in experimental groups. Damaged area (%) of gastric mucosa was determined using ImageJ software. (**C**) For histopathological inspection, H&E-stained gastric mucosal sections were observed under an Olympus BX53 microscope (original magnification × 100). The pathological score for epithelial cell loss, haemorrhage, and erosion was randomly quantified on an H&E-stained section from each experimental group (*n* = 5). Results are presented as mean ± SD of five independent experiments. ^###^ *p* < 0.001 vs. the CON group; * *p* < 0.05, ** *p* < 0.01, *** *p* < 0.001 vs. the INDO group. ^$^ *p* < 0.05, ^$$^ *p* < 0.01 vs. the CH-treated group. CON, control group; INDO, indomethacin-induced rats (80 mg/kg, p.o.); STI, INDO-induced rats with Stillen^®^ (100 mg/kg, p.o.); CB, INDO-induced rats with cabbage (4.5 g/kg, p.o.); CH, INDO-induced rats with chestnut honey (0.5 g/kg, p.o.); CH + CB, INDO-induced rats with mixture of CH and CB (5 g/kg, p.o.).

To investigate whether the anti-inflammatory action of the CH and CB mixtures also applies to the stomach, the protective effects of CH and CB mixtures were evaluated against INDO-induced gastric damage in rats (Figure 4A). First, CH and CB mixtures were manufactured in various ratios, such as 1:9, 1:1, and 9:1, to compare the efficacy differences based on the mixing ratio of CH and CB. Macroscopic examination of the gastric mucosa indicated that the damaged area was significantly escalated by INDO administration and was reduced by the administration of STI, CB, CH, and CH + CB mixtures (Figure 4B and Figure S2). Histopathological examinations of gastric sections also revealed that the scores for epithelial cell loss, haemorrhage, and erosion—which were significantly higher after INDO administration—were reduced by the administration of STI, CB, CH, and CH + CB mixtures (Figure 4C). These results indicate the significant effectiveness of CH + CB 9:1 administration, thereby suggesting that a higher mixing ratio of CH improves the protective effects against gastric damage.

### 3.6. KACH and CB Mixtures Protect against Gastric Damage in INDO-Induced Rats

**Figure 5:**
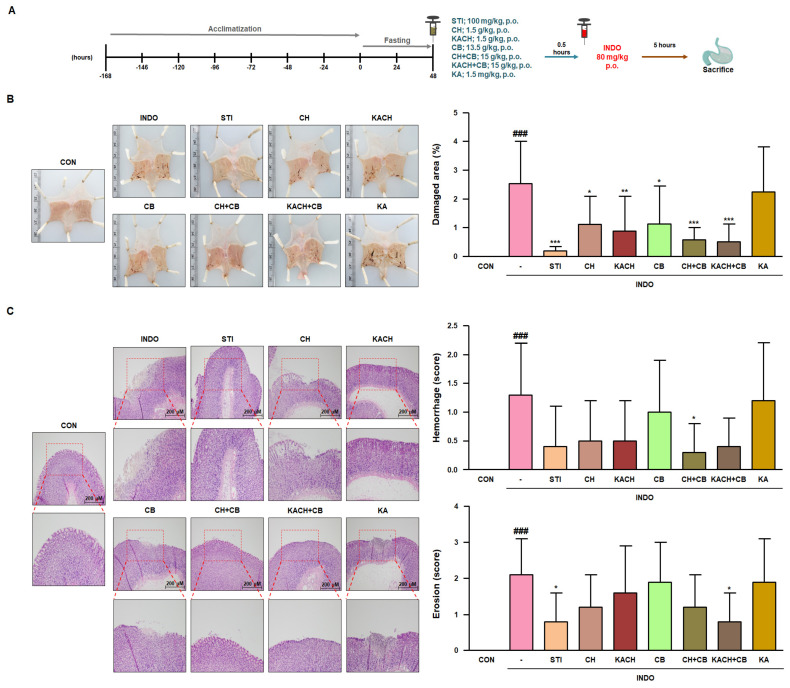
The protective effect of KACH and CB mixtures on gastric damage in INDO-induced rats. (**A**) Schematic diagram of the second animal experiment. (**B**) Visual inspection of gastric lesions in experimental groups. Damaged area (%) of gastric mucosa was determined using ImageJ software. (**C**) For histopathological inspection, H&E-stained gastric mucosal sections were observed under an Olympus BX53 microscope (original magnification × 100). The pathological score for haemorrhage and erosion was randomly quantified on an H&E-stained section from each experimental group (*n* = 8). Results are presented as mean ± SD of eight independent experiments. ^###^ *p* < 0.001 vs. the CON group; * *p* < 0.05, ** *p* < 0.01, *** *p* < 0.001 vs. the INDO group. CON, control group; INDO, indomethacin-induced rats (80 mg/kg, p.o.); STI, INDO-induced rats with Stillen^®^ (100 mg/kg, p.o.); CH, INDO-induced rats with chestnut honey (1.5 g/kg, p.o.); KACH, INDO-induced rats with kynurenic acid increased CH (1.5 g/kg, p.o.); CB, INDO-induced rats with cabbage (13.5 g/kg, p.o.); CH + CB, INDO-induced rats with mixture of CH and CB (15 g/kg, p.o.); KACH + CB, INDO-induced rats with mixture of KACH and CB (15 g/kg, p.o.); KA, INDO-induced rats with kynurenic acid (1.5 mg/kg, p.o.).

To further validate the effect of CH in INDO-induced rats, KACH with increased content of KA, the active ingredient of CH, was manufactured and a secondary experiment was conducted using KACH and its mixture (Figure 5A). Visual inspection revealed that the distinct hemorrhagic lesions of the gastric mucosa observed in the INDO group were alleviated in the STI, CH, KACH, CB, CH + CB, and KACH + CB groups. In particular, the damaged area of total gastric mucosal area, which accounted for 2.53% ± 1.48 in the INDO group, decreased significantly to 0.58% ± 0.43 and 0.52% ± 0.61 in the CH + CB and KACH + CB groups, respectively (Figure 5B and Figure S3). Histopathological examination of the gastric sections revealed severe haemorrhage and erosion in the gastric mucosa of the INDO group when compared to the CON group, which showed a normal histological structure. However, this effect was reduced by CH + CB or KACH + CB administration (Figure 5C). Therefore, these results indicate that the protective effect against gastric mucosal damage is improved in CH + CB and KACH + CB groups compared to that of the CH group, suggesting that the mixture of KACH and CB is more effective.

### 3.7. KACH and CB Mixtures Suppress the Inflammatory Response in INDO-Induced Rats

**Figure 6:**
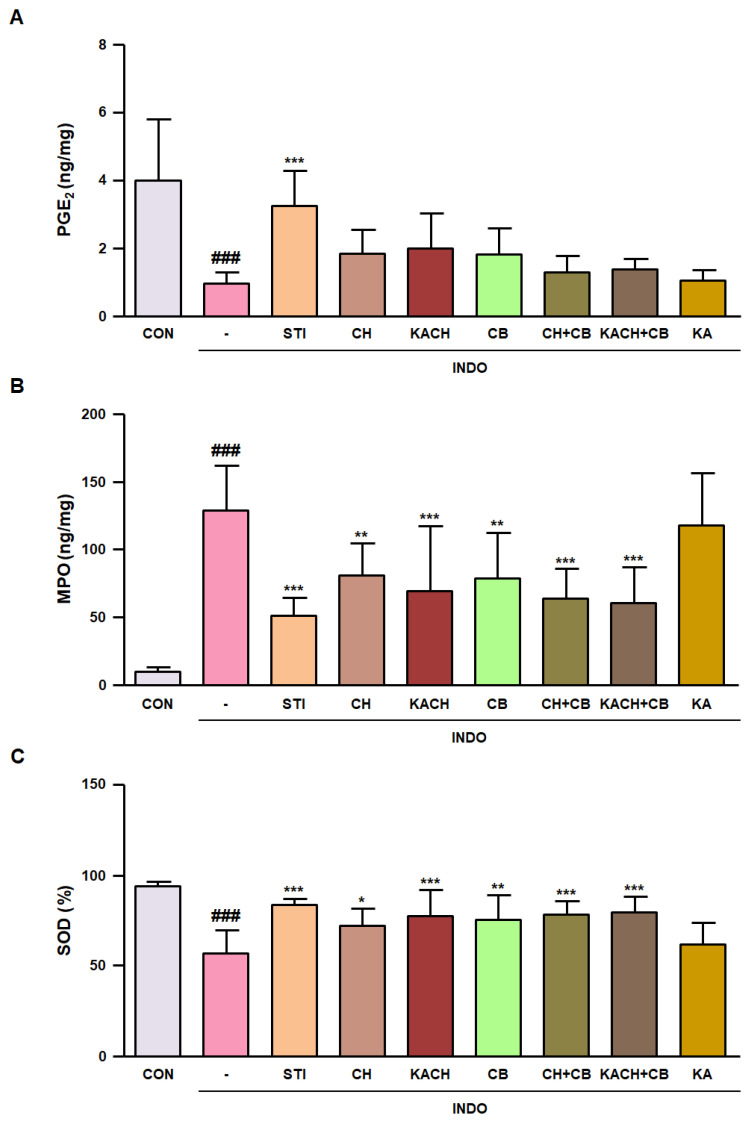
Regulatory effect of KACH and CB mixtures on the level of PGE_2_, MPO, and SOD in INDO-induced rats. (**A**–**C**) The PGE_2_, MPO, and SOD levels in gastric mucosal tissue were determined using commercial ELISA kits. Results are presented as mean ± SD of eight independent experiments. ^###^ *p* < 0.001 vs. the CON group; * *p* < 0.05, ** *p* < 0.01, *** *p* < 0.001 vs. the INDO group. CON, control group; INDO, indomethacin-induced rats (80 mg/kg, p.o.); STI, INDO-induced rats with Stillen^®^ (100 mg/kg, p.o.); CH, INDO-induced rats with chestnut honey (1.5 g/kg, p.o.); KACH, INDO-induced rats with kynurenic acid increased CH (1.5 g/kg, p.o.); CB, INDO-induced rats with cabbage (13.5 g/kg, p.o.); CH + CB, INDO-induced rats with mixture of CH and CB (15 g/kg, p.o.); KACH + CB, INDO-induced rats with mixture of KACH and CB (15 g/kg, p.o.); KA, INDO-induced rats with kynurenic acid (1.5 mg/kg, p.o.).

To investigate whether the KACH and CB mixtures improved the levels of PGE_2_—a gastric mucosal defensive factor—ELISA was performed using gastric tissues from INDO-induced rats. PGE_2_ levels reduced by INDO administration were restored by sample administration but were not significant, except for the positive control STI (Figure 6A). The level of MPO, an indicator of neutrophil activation used to evaluate inflammatory responses, was significantly elevated following INDO administration. However, it was significantly suppressed in the sample administration groups, except for KA (Figure 6B). Furthermore, the SOD levels were significantly suppressed by INDO administration compared to those in the CON group and were increased by the administration of samples other than KA (Figure 6C). These results indicate that the combination of CH or KACH with CB more effectively reduced the inflammatory and oxidative stress responses contributing to gastric mucosal damage compared to CH alone.

## 4. Discussion

Gastritis, an acute or chronic inflammatory response that damages the gastric mucosa, remains a major public health problem because it is difficult to heal and recurs easily [24]. Because gastritis is also caused by NSAIDs such as INDO, which are widely used clinically to treat inflammation, the use of dietary supplements or natural products that have anti-inflammatory activity with few side effects is considered [25]. CB, which is rich in SA with anti-inflammatory properties, is beneficial in treating various gastrointestinal and digestive disorders but has the disadvantage of having a strong bitter taste [26,27]. CH, the only food rich in KA, which is a metabolite of tryptophan, has many health benefits and has long been used as an instant energy source to improve both the bitterness and efficacy of medicinal plants [28]. From this perspective, mixing CH with CB—which tastes bitter but is widely known for its gastroprotective effect—is expected to elicit a positive response. Furthermore, considering the anti-inflammatory activity of KA reported in previous studies [29], it was expected that increasing the CH mixing ratio or KA content in CH would improve the effects of CH and CB mixtures. Accordingly, this study not only confirmed the effect of CH + CB in various proportions but also the effect of KACH + CB to determine the synergistic effect of CH and CB mixtures in LPS-stimulated RAW 264.7 macrophages and INDO-induced rats.

Since excessive inflammatory responses and oxidative stress result in damage to the gastric mucosa, antioxidants can be an important alternative for the treatment of gastritis [30]. Macrophages with a high sensitivity to the initiators of inflammation and oxidative stress, such as LPS, are considered essential immune cells for evaluating antioxidant activity [31]. In activated macrophages, NF-κB enters the nucleus by the degradation of IκB-α through phosphorylation of IKK-α/β, thereby enhancing the levels of inflammation-related molecules [32]. The current study found that CH + CB or KACH + CB inhibit the levels of proinflammatory cytokines by preventing the transcriptional activity of NF-κB p65 in LPS-stimulated RAW 264.7 macrophages than in a single treatment. This indicates that CH + CB or KACH + CB, which contain antioxidant-rich SA and KA, can alleviate gastritis by exerting strong anti-inflammatory effects.

Nrf2, which neutralizes ROS by activating endogenous antioxidant enzymes, reduces inflammatory responses by blocking the transcriptional activity of NF-κB [33]. Under normal conditions, the Nrf2/Keap1 complex, deactivated in the cytoplasm, is separated by oxidative stress, which resists intracellular oxidative stress by inducing the nuclear translocation of Nrf2 [34]. This study confirmed that CH + CB or KACH + CB increase the expression of antioxidant enzymes, including HO-1, SOD, GPx, and GSTs by promoting the nuclear translocation of Nrf2 in LPS-stimulated RAW 264.7 macrophages more effectively than in a single treatment. Consistent with this, we confirmed that either CH + CB or KACH + CB decreased MPO activity by increasing SOD activity in the damaged gastric mucosa of INDO-induced rats. This suggests that either CH + CB or KACH + CB alleviated gastric mucosal damage by activating antioxidant systems that neutralize oxidative stress, which was also effective at low-dose administration. However, KA administration at the corresponding dose did not significantly affect INDO-induced gastric mucosal damage. These results imply that the effect of CH is exerted through complex interactions with other active ingredients.

In summary, these investigations demonstrate that the potent anti-inflammatory action of the CH and CB combination can prevent gastritis by reducing bleeding and oedema of the mucosal layer, which is particularly effective in the KACH and CB combination. However, further studies should be conducted to identify specific targets of CH + CB and KACH + CB, such as NF-κB and Nrf2, to gather additional information for future clinical applications.

## 5. Conclusions

To our knowledge, this study is the first to demonstrate that both CH + CB and KACH + CB not only enhance anti-inflammatory activity but also alleviate gastric mucosal damage. Mechanistically, CH + CB or KACH + CB upregulated the expression of antioxidant enzymes by activating Nrf2, which ultimately suppressed the inflammatory response in LPS-stimulated RAW 264.7 macrophages. Moreover, CH + CB and KACH + CB enhanced SOD levels and suppressed MPO levels, ultimately protecting INDO-induced gastric mucosal damage in rats. Therefore, these results show that both CH + CB and KACH + CB have the potential as functional health foods that can be used to prevent or alleviate gastric mucosal damage caused by NSAIDs.

## Figures and Tables

**Table 1:** Information on mouse oligonucleotide primers.

Gene	Forward Primer (5′-3′)	Reverse Primer (5′-3′)
IL-6	TTCCATCCAGTTGCCTTCTTG	GGGAGTGGTATCCTCTGTGAAGTC
TNF-α	ATGAGCACAGAAAGCATGAT	TACAGGCTTGTCACTCGAAT
HO-1	GAATGAACACTCTGGAGATGACAC	TGTGAGGGACTCTGGTCTTTG
SOD1	TGAAAGCGGTGTGCGTGCTGAAG	GGAATGCTCTCCTGAGAGTGAGA
GPx1	ACACCGAGATGAACGATCTG	ATGTACTTGGGGTCGGTCAT
GSTM1	CACAAGATCACCCAGAGCAA	TGGTTCTCCACAATGTCTGC
GSTP1	GCCCAGATGGATATGGTGAA	ATGGGACGGTTCACATGTTC
β-actin	CCACACCTTCTACAATGAGC	CACAGGATTCCATACCCAAG

## Data Availability

The datasets used and/or analyzed in the current study are available from the corresponding author upon reasonable request.

## Supplementary Materials

The following supporting information can be downloaded at: https://www.mdpi.com/article/10.3390/nu16030389/s1, Figure S1: HPLC-VWD analysis of MCHCB 1–4; Figure S2. Histological analysis of first animal experiment; Figure S3. Histological analysis of second animal experiment; Table S1: Calibration curves of standards KA and SA; Table S2: Content of standards KA and SA in MCHCB 1–4.
