# Supplementary material for: Anti-Inflammatory Effect of Chestnut Honey and Cabbage Mixtures Alleviates Gastric Mucosal Damage
Source: Nutrients. 2024 Jan 29;16(3):389. doi: 10.3390/nu16030389 (PMC10857084; doi:10.3390/nu16030389)
Supplement: Supplementary file 1 [file nutrients-16-00389-s001.zip › nutrients-2781157-supplementary.pdf]

Table S1. Calibration curves of standards KA and SA.

Compound	t_R	Range (mg/mL)	Calibration equation	r^2
KA	12.8	0.0625 – 1.0000	Y = 35.518X + 656.1	0.9990
SA	22.2	0.0009 – 0.0156	Y = 37.317X + 8.0685	0.9992

KA, kynurenic acid; **SA**, sinapic acid; **Y**, peak area; **X**, concentration of standards (μg/mL); r^2, correlation coefficient based on three data points in the calibration curves.

Table S2. Content of standards KA and SA in MCHCB 1–4.

Sample	KA Content (mg/g mix.)	SA Content (mg/g mix.)
MCHCB 1	2.77 ± 0.01	ND
MCHCB 2	ND	0.006 ± 0.001
MCHCB 3	0.21 ± 0.01	0.007 ± 0.001
MCHCB 4	0.44 ± 0.01	0.015 ± 0.001

KA, kynurenic acid; **SA,** sinapic acid; **MCHCB,** chestnut honey (CH) and cabbage (CB) single or in combination; **MCHCB 1**, CH; **MCHCB 2**, CB; **MCHCB 3**, CH+CB 1:9; **MCHCB 4**, kynurenic acid increased CH+CB 1:9; **ND,** not detected.

Figure S1

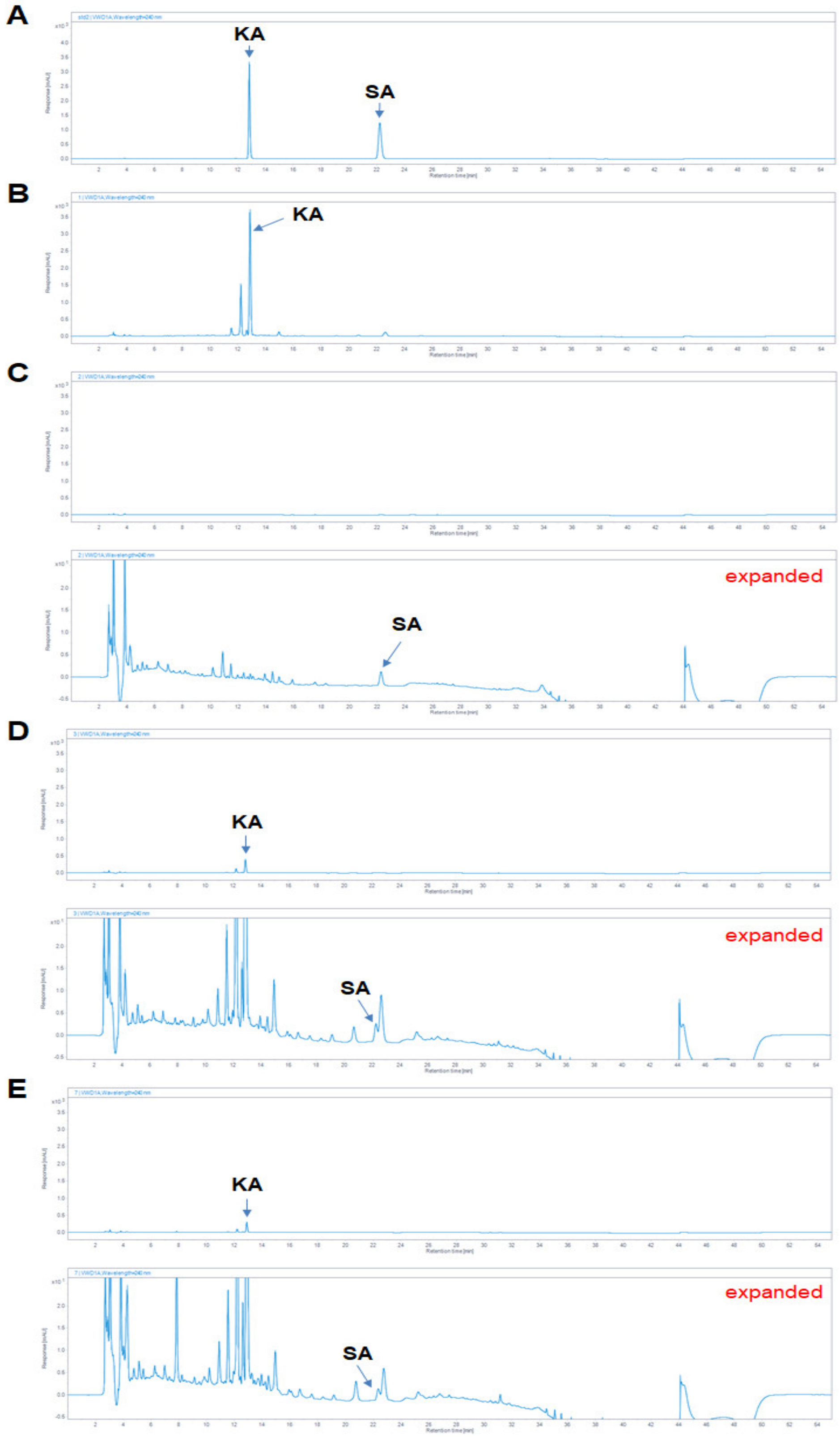

Figure S1. HPLC-VWD analysis of MCHCB 1–4. Infographic for HPLC-VWD analysis of (**A**) the standard (KA and SA) and (**B**–**E**) MCHCB 1–4. **MCHCB,** chestnut honey **(**CH**)** and cabbage **(**CB**)** single or in combination; **MCHCB 1**, CH; **MCHCB 2**, CB; **MCHCB 3**, CH+CB 1:9; **MCHCB 4**, kynurenic acid increased CH+CB 1:9; **KA**, kynurenic acid; **SA**, sinapic acid.

Figure S2

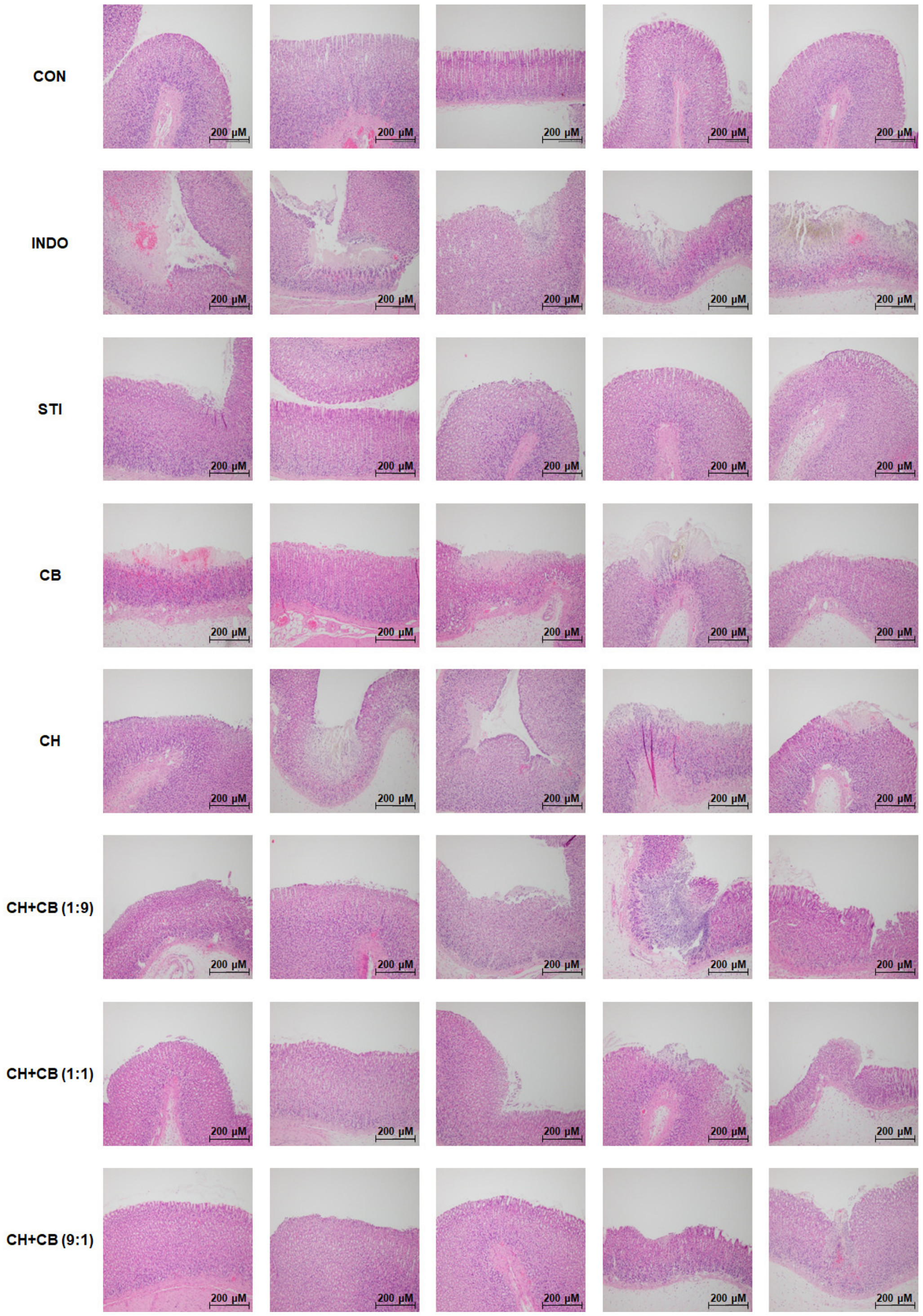

Figure S2. Histological analysis of first animal experiment. For histopathological inspection, H&E-stained gastric mucosal sections were observed under an Olympus BX53 microscope (original magnification x 100, n = 5). **CON**, control group; **INDO**, indomethacin-induced rats (80 mg/kg, p.o.); **STI**, INDO-induced rats with Stillen® (100 mg/kg, p.o.); **CB**, INDO-induced rats with cabbage (4.5 g/kg, p.o.); **CH**, INDO-induced rats with chestnut honey (0.5 g/kg, p.o.); **CH+CB**, INDO-induced rats with mixture of CH and CB (5 g/kg, p.o.).

Figure S3

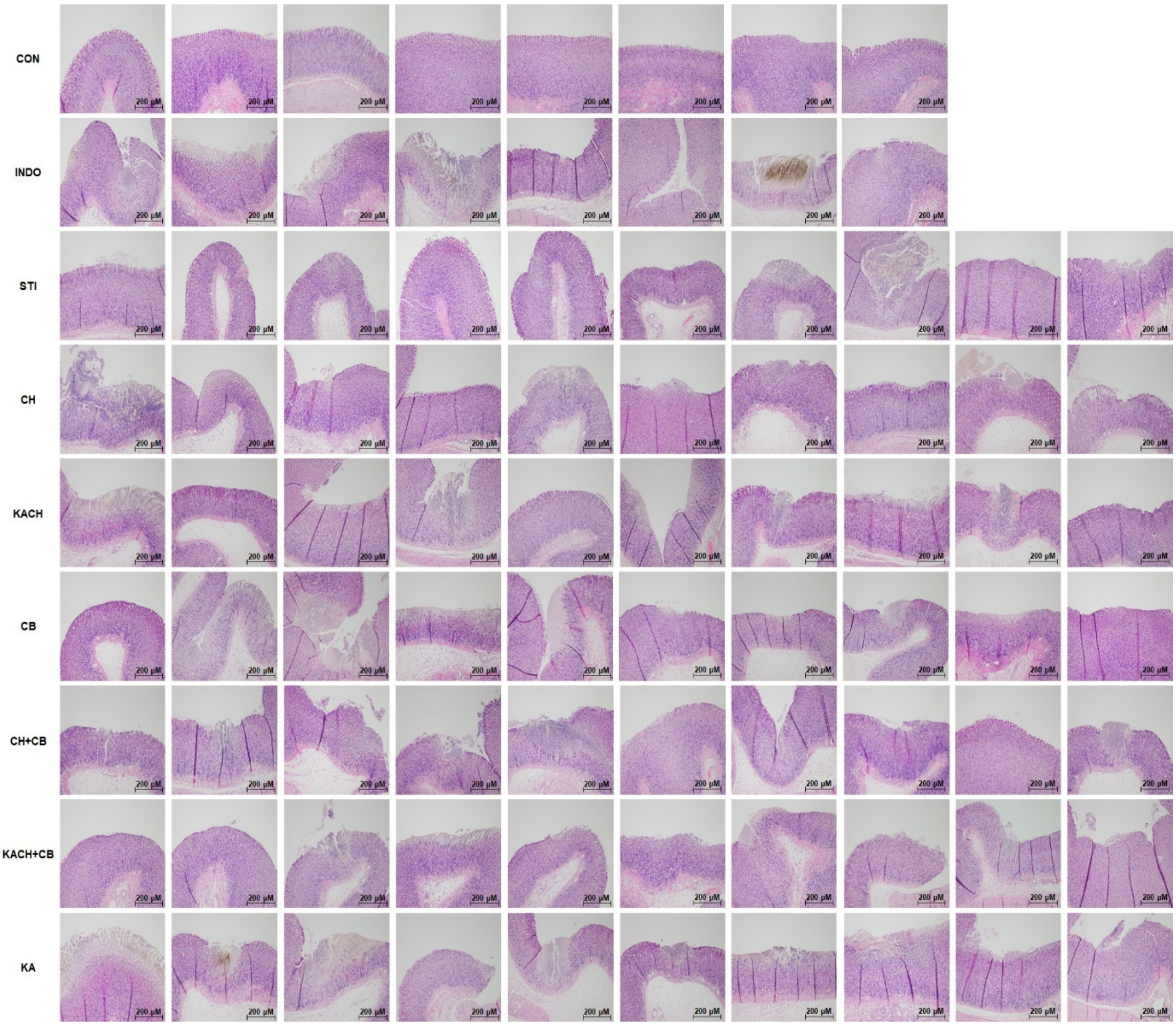

Figure S3. Histological analysis of second animal experiment. For histopathological inspection, H&E-stained gastric mucosal sections were observed under an Olympus BX53 microscope (original magnification x 100, n = 8-10). **CON**, control group; **INDO**, indomethacin-induced rats (80 mg/kg, p.o.); **STI**, INDO-induced rats with Stillen® (100 mg/kg, p.o.); **CH**, INDO-induced rats with chestnut honey (1.5 g/kg, p.o.); **KACH**, INDO-induced rats with kynurenic acid increased CH (1.5 g/kg, p.o.); **CB**, INDO-induced rats with cabbage (13.5 g/kg, p.o.); **CH+CB**, INDO-induced rats with mixture of CH and CB (15 g/kg, p.o.); **KACH+CB**, INDO-induced rats with mixture of KACH and CB (15 g/kg, p.o.); **KA**, INDO-induced rats with kynurenic acid (1.5 mg/kg, p.o.).
